# Polyoxometalate-based microcrystal arrays patterned on air-grid superwettable surface

**DOI:** 10.1038/s41598-018-32279-4

**Published:** 2018-09-17

**Authors:** Tianzhan Zhang, Yuefeng Wang, Jun-Bing Fan, Jingxin Meng, Yangguang Li, Enbo Wang, Shutao Wang

**Affiliations:** 1 CAS Key Laboratory of Bio-inspired Materials and Interfacial Science, CAS Center for Excellence in Nanoscience, Technical Institute of Physics and Chemistry, Chinese Academy of Sciences, Beijing, 100190 P. R. China; 2 College of Material Science and Engineering, Jilin Jianzhu University, Changchun, 130118 P. R. China; 3 University of Chinese Academy of Sciences, Beijing, 100049 P. R. China; 4 Key Laboratory of Polyoxometalate Science of Ministry of Education, Faculty of Chemistry, Northeast Normal University, Changchun, 130024 P. R. China

## Abstract

Surface patterning of polyoxometalates (POMs) is an important step to gain functional materials and devices. However, some special requirements such as complex operation steps or strict synthesis environment greatly limit their further applications. Herein, we have employed a simple and universal strategy for patterning POM-based microcrystal arrays on air-grid superwettable surfaces. The size and distribution of POM crystals were precisely adjusted by varying the pillar parameter of superwettable surface and concentration of POM mother liquid. We envision that this POM patterning method may bring valuable insights for designing POM-based functional materials and devices.

## Introduction

Polyoxometalates (POMs), as a class of discrete anionic metal oxides, attract increasing interest and concern because their diverse molecular structures and unique physicochemical properties make them ideal functional materials for a large domain of applications^1–7^ such as inhibiting Alzheimer’s disease^8^, removing multiple contaminants^9^, refrigerating for ultra-low temperatures^10^ as well as splitting water through visible or UV light^11–15^. In most cases, precisely patterned POMs on the surface are pre-requisites that could accomplish a high degree of spatial control leading to many unique applications. For example, through electrostatic forces, Preyssler-typed POM (i.e., [Eu(H_2_O)P_5_W_30_O_110_]^12-^) were readily adsorbed to prepare electrochromic devices, which show sufficiently high optical contrast^16^. Through the formation of amide bond, Anderson-typed POM clusters (i.e., MnMo_6_O_24_) can be covalently grafted to fabricate patterned surfaces, which show strong adhesive response between human fibroblasts and POM areas^17^. However, some special requirements for modifying POM components onto surfaces such as complex operation steps (i.e., layer-by-layer methods), possibly limit their further applications. Therefore, a question may rise whether we can find a simple and universal method for patterning POM materials on the surfaces.

As well known, one of the important aspects of POM clusters is their high solubility in a variety of solvents, which can be readily prepared into uniform mother liquids for patterning POM materials through solution processing methods. However, commonly used solution evaporation method often lead to the failure in controlling the nucleate position because the continuous solution liquid film would break into random liquid domains in evaporation process. To solve this problem, many methods such as dip coating^18^, inkjet printing^19^ and superwettable pattern^20–23^ have been developed to obtain patterned materials. More recently, the air-grid superwettable surfaces (e.g., superhydrophobic surfaces)^24^ are employed as templates in growing crystal arrays from aqueous solutions because the trapped air act as separating barriers to reduce the defect and non-uniformity of microcrystal patterning^25^. For instance, McCarthy *et al*. fabricated controllable NaCl crystal arrays with the assistance of superhydrophobic surface *via* a simple dip-coating process^26^. Simultaneously, Jiang and co-workers developed a technology for microcrystal patterning involving “clinging microdroplets”, revealing that superhydrophobic pillar-structured surfaces with high liquid-solid adhesion can be used to form microarrays including NaCl crystals, protein, microsphere, and nanoparticle aggregates^27^. Then, Hatton and Aizenberg also patterned CaCO_3_ crystals deposition using a superhydrophobic post-structured surface, which can be utilized for the heterogeneous nucleation and localized growth of CaCO_3_ crystals from solution and avoiding nonspecific adsorption^28^. In addition, some single-crystalline structures with excellent optoelectrical properties have recently been reported such as microplates^29^, wires^30,31^ and belts^32^. Therefore, we wonder whether this unique air-grid superwettable surface can be employed for fabricating periodic POM microcrystal arrays?

Herein, we demonstrate that patterned POM microcrystal arrays can be prepared by precisely controlling the dewetting process of POM mother liquid on superhydrophobic pillar-structured template. Through the unique capillary force and adhesion force, the dewetting behavior of POM mother liquid can be precisely regulated, thereby facilitating the formation of individual microdroplets on the top of each pillar with homogenous size and specific location. Ultimately, these microdroplets can be transformed to pattern POM crystal arrays with uniform size and precise location. Through tailoring the diameter of pillars or the concentration of POM mother liquid, the size of the as-prepared POM microcrystals might be readily tunable. While the distribution and density of POM crystals can be adjusted by simply tuning the spacing of pillars. Therefore, it is anticipated that we provide a simple and universal strategy to fabricate patterned POM crystal arrays, which probably bring new insights in designing POM-based function materials and devices for various applications.

## Results and Discussion

### The formation process of POM crystals upon the air-grid superwettable surface

**Figure 1:**
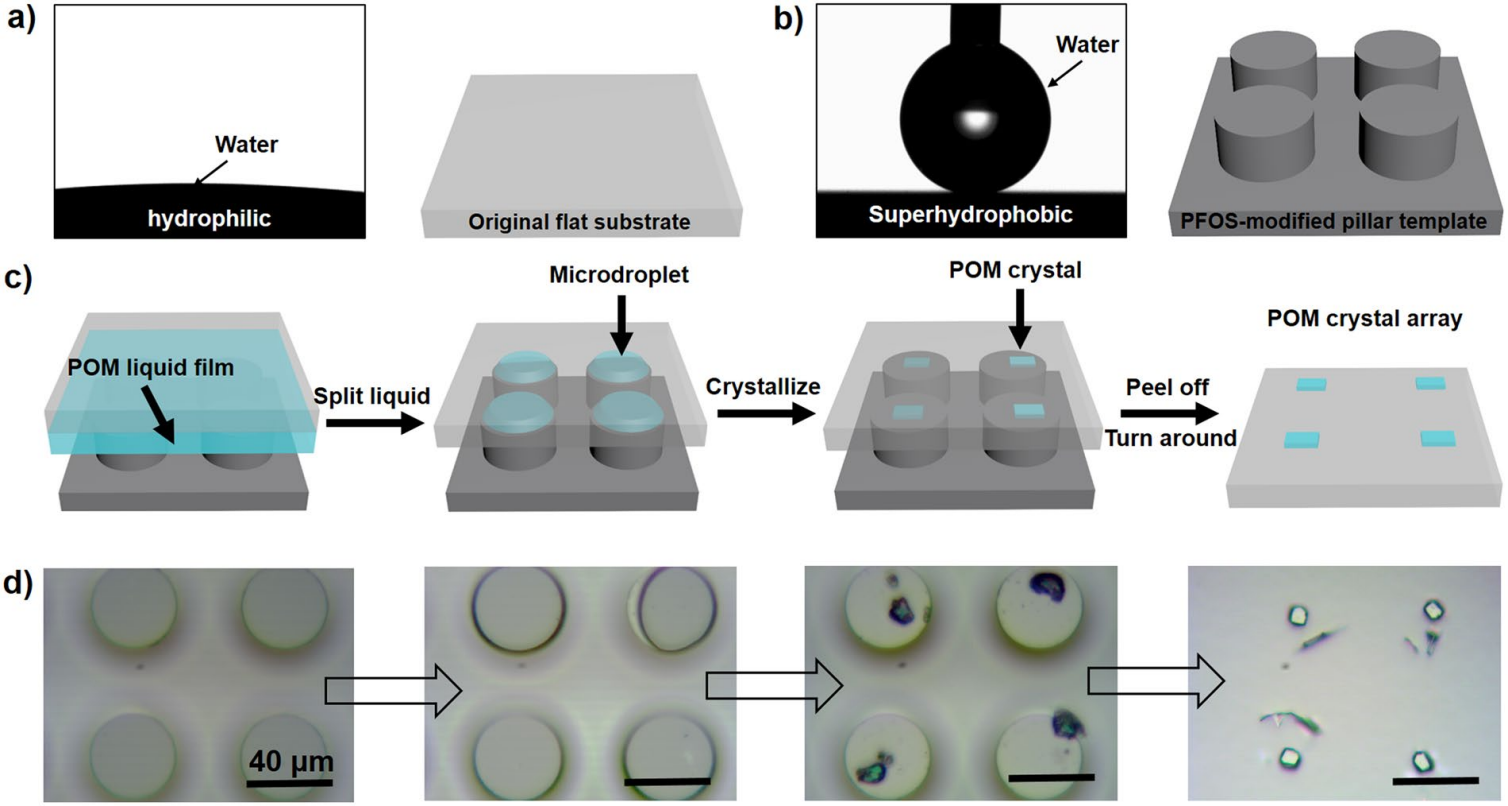
The dewetting process of POM mother liquid on air-grid superwettable surface. The water contact angles (left) and scheme view (right) of employed substrates for fabricating POM crystal arrays, including (**a**) original flat substrate with hydrophilic property and (**b**) PFOS-modified pillar template with superhydrophobic property. (**c**) Schematic illustration and (**d**) corresponding optical images of *in situ* dewetting process of POM mother liquid on air-grid superwettable surface. Firstly, a thin POM liquid film can be observed between hydrophilic flat substrate and superhydrophobic pillar-structured template. Then, individual POM microdroplet can be observed on the top of each pillar (black circle) by splitting the continuous liquid film through air pocket between pillars. With the further shrinkage of liquid, POM crystals could be concentrated from these microdroplets. After gently removing pillar-structured template, the patterned POM crystal arrays could be transferred onto the flat substrate. Scale bars, 40 µm.

To reveal the whole dewetting process on this air-grid superwettable surface, we observed *in situ* evaporation performance of POM mother liquid in an optical microscope. In detail, two kinds of silicon substrates, that is, original flat substrate and PFOS-modified pillar-structured substrate were employed to prepare patterned POM microcrystal arrays. Original flat substrate was hydrophilic with a water contact angle (CA) of 7 ± 1° (Fig. 1a), while PFOS-modified pillar-structured substrate was superhydrophobic with a CA of 162 ± 2° (Fig. 1b). A classical Anderson-typed POM component, (NH_4_)_3_[CoMo_6_O_24_H_6_] (denoted as CoMo_6_), was selected as an example for the preparation of POM mother liquid. The scheme and corresponding optical images clearly revealed the whole dewetting process of POM mother liquid (Fig. 1c,d). In brief, a droplet of CoMo_6_ mother liquid (2 mM, ca. 25 μL) is firstly dropped onto a hydrophilic flat glass substrate. To directly observe the dewetting process in an upright microscope, a superhydrophobic pillar-structured template is covered by the hydrophilic substrate, forming a sandwich-typed system. Then, with gradual evaporation of water in POM liquid, unique capillary force and adhesion force precisely guide the dewetting behavior of POM mother liquid, thereby facilitating the formation of regularly individual microdroplets on the top of each pillar. On one hand, a plenty of air pockets trapped in the pillar gaps can split the liquid into regularly individual microdroplets owing to the hydrophobic nature of pillar sidewalls. On the other hand, the liquid film pin upon the pillar tops, due to the high adhesion of the pillar tops. Later, microcrystal arrays with homogenous size and restricted position can be patterned from these regularly individual microdroplets with the further evaporation of water. Finally, the well-defined microcrystal arrays are transferred onto the flat substrate after gently removing the pillared template. The chemical components of initial powder and assembled microcrystals can be confirmed by their IR spectra in Fig. S1, which closely coincided with typical peaks of POM components (e.g., CoMo_6_)^33^. Therefore, the air-grid superwettable surface can be employed to pattern POM microcrystal arrays, showing excellent controllability and easy accessibility.

### Tailoring the size and distribution density of the patterning

**Figure 2:**
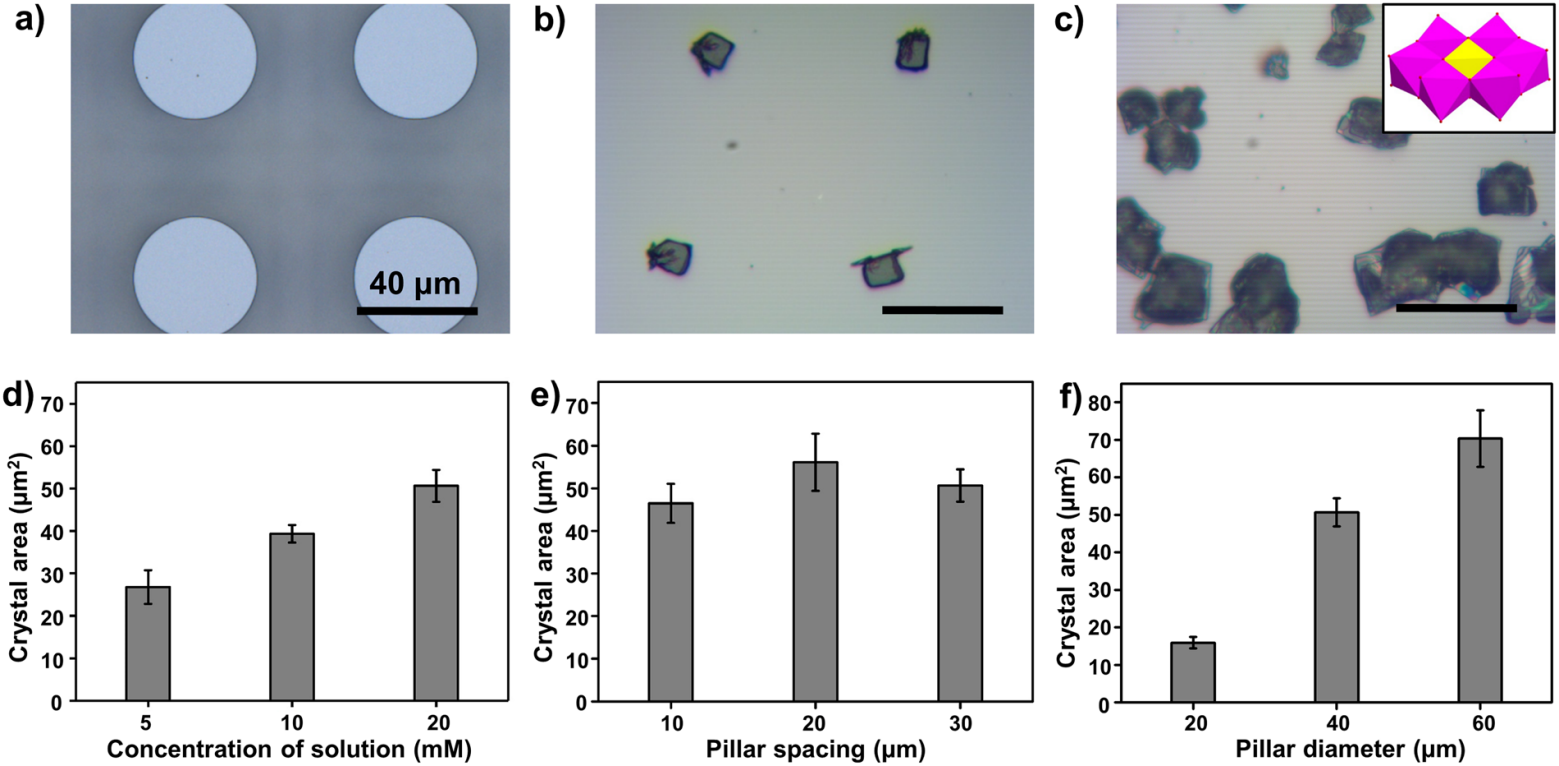
The influence of different factors including the concentration of POM mother liquid, the spacing and diameter of pillar on patterning POM crystal arrays. (**a**) The optical images of superhydrophobic pillar-structured template and (**b**) corresponding POM crystal arrays. (**c**) The random large crystals prepared on flat substrate by employing conventional solution evaporation method. Insert represents the crystal structure of Anderson-typed POM, (NH_4_)_3_[CoMo_6_O_24_H_6_] (denoted as CoMo_6_). (**d**) The crystal areas increased with the enhancement of POM solution concentrations. (**e**) The crystal areas still kept regardless of the pillar spacings. (**f**) With the same concentration of POM solution, the crystal areas increased with the enlargement of pillar diameters. Scale bars, 40 µm.

When employing the above-mentioned air-grid superwettable surface (Fig. 2a), regular POM microcrystal arrays can be fabricated in the crystallization process (Fig. 2b). On the contrary, the dewetting process of POM mother liquid is often uncontrollable when using conventional solution-processing methods. Figure 2c shows that large area of POM polycrystals can be fabricated with uncontrollable sizes and random positions, suggesting that the continuous POM liquid film on original flat substrate would break into random liquid domains. To realize the regulation of crystal arrays, we firstly investigated the influence of concentrations of POM mother liquid. Taking Anderson-typed POM (i.e., CoMo_6_, insert in Fig. 2c) as an example, the superhydrophobic silicon templates with pillar spacing of 30 µm and pillar diameter of 40 µm were used to prepare CoMo_6_ crystal arrays. Figure 2d showed that CoMo_6_ crystal sizes (e.g., crystal areas) changed from 26.8 ± 4.0 to 50.7 ± 3.8 μm^2^ when the concentrations of the CoMo_6_ solution increased from 5 to 20 mM, indicating the increased value of crystal area following the enlarged the concentrations of CoMo_6_ solution. Therefore, these results indicated that the concentration of POM solution could be utilized to mediate the size of POM crystals.

Furthermore, we explore the influence of the spacing and diameter of pillars on patterning POM crystal arrays. To understand the influence of pillar spacing, we designed three kinds of pillar-structured templates with similar pillar diameter (i.e., 40 µm) and pillar height (i.e., 20 µm) but different pillar spacings (i.e., 10, 20 and 30 µm) (Fig. S2a). Taking CoMo_6_ as an example, the optical images in Fig. 2e showed that the position of POM crystals can be readily adjusted by tailoring the pillar spacings. However, the crystal sizes were not significantly changed with the alteration of pillar spacings. As shown in Fig. S3a, the distribution density of crystal arrays obviously decrease with the increase of pillar spacings. So we conclude that the pillar spacing can be used for the change of the crystal distribution density rather than that of crystal size. To reveal the influence of pillar diameter, we fabricated three types of pillar-structured silicon templates with the same pillar spacing of 30 µm and pillar height of 20 µm, but different pillar diameters of 20, 40 and 60 µm (Fig. S2b). Figures 2f and S3b both showed that crystal sizes became larger with the enhancement of pillar diameters, suggesting that larger pillars can hold larger droplets and thereby producing larger POM microcrystals. Therefore, these results demonstrate that pillar spacing can be dominated for the distribution of POM crystals, while pillar diameter can be tailored for controlling crystal sizes.

### The crystallization behaviors of POM on silicon substrates with different wettability

**Figure 3:**
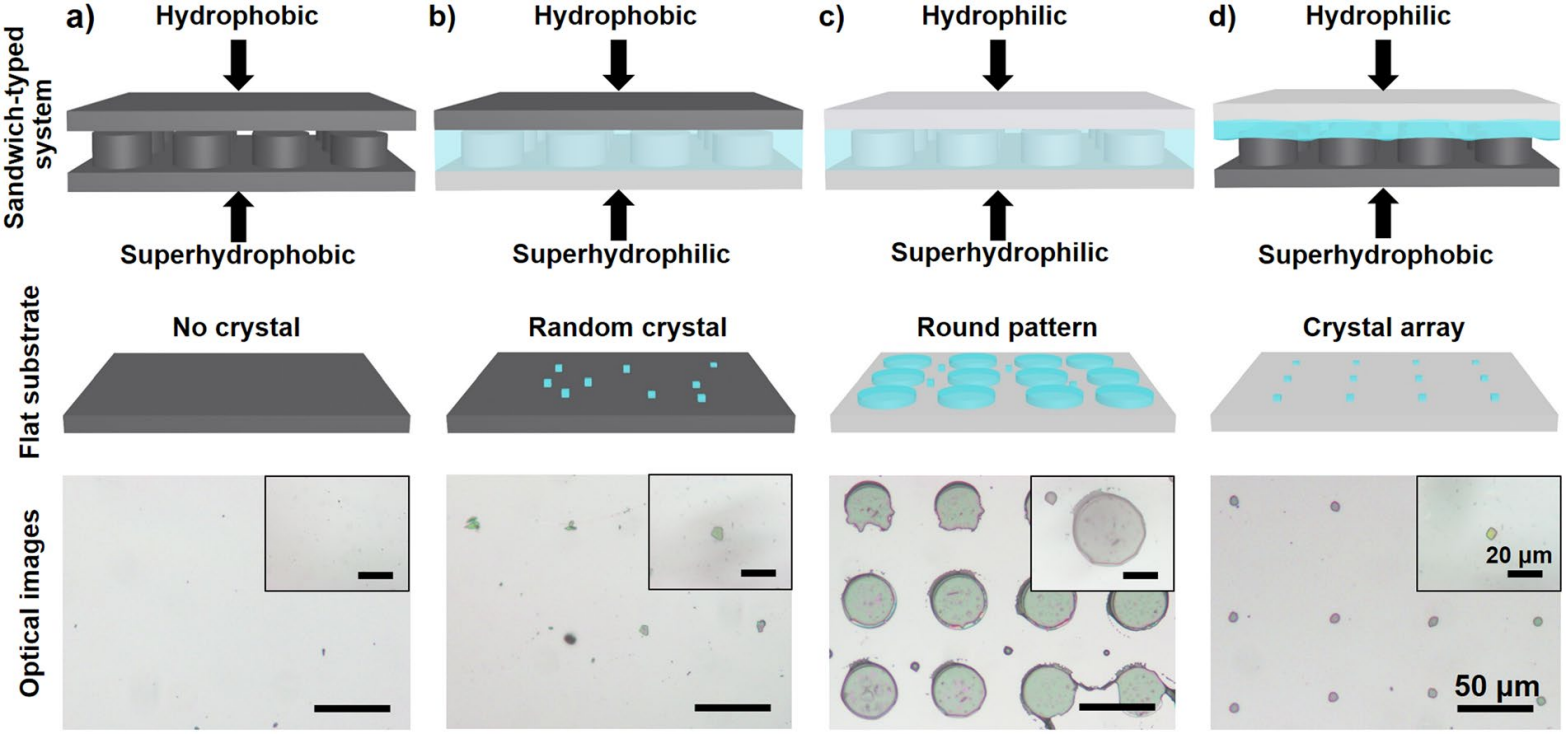
Schematic illustration of different sandwich-typed systems (top), POM components assembled on flat substrates (middle), and corresponding optical images (bottom). (**a**) For hydrophobic flat substrate and superhydrophobic pillar-structured template, the solution is completely extruded from the interspace of two substrates, revealed by no obvious crystals on the flat substrate; (**b**) For hydrophobic flat substrate and superhydrophilic pillar-structured template, solution mainly fill in the spacing of superhydrophilic pillars but rarely rest on the top of these pillars, which can be demonstrated by a few disordered crystals; (**c**) For hydrophilic flat substrate and superhydrophilic pillar-structured template, POM solution fills in the top and spacing of the pillars, verified by the rounded and disordered patterns; (**d**) For hydrophilic flat substrate and superhydrophobic pillar-structured template, well-ordered POM crystal arrays can be observed because the air pocket efficiently restricts the spreading of solution and is benefit for the generation of microdroplets and subsequent POM crystals. Scale bars, 50 µm; scale bars of insert images, 20 µm.

To deeply explore the influence of surface wettability, we designed four kinds of sandwich-typed systems (Fig. 3) by employing silicon substrates with different wettabilities including hydrophilic and hydrophobic flat substrate, superhydrophobic and superhydrophilic pillar-structured template (Figs 1 and S4). For the combination of hydrophobic flat substrate and superhydrophobic pillar-structured template (Fig. 3a), there was no obvious crystals on the flat substrate, probably indicating that POM mother liquid was extruded by the air trapped in the interstices of the micropillars. When we employed the combination of hydrophobic flat substrate and superhydrophilic pillar-structured template (Fig. 3c), a few disordered crystals indicated that POM mother liquid rarely rested on the top of these pillars, but mainly fill in the spacing of superhydrophilic pillars for their adhesive properties. For the combination of hydrophilic flat substrate and superhydrophilic pillar-structured template (Fig. 3c), POM mother liquid could fully cover the top and interspaces of pillars owing to higher adhesive force between superhydrophilic substrate and POM mother liquid. As a result, the morphology of crystals were controlled by the top shape of superhydrophilic pillar, showing rounded POM crystals and few disordered particles on the hydrophilic flat substrate. As shown in Fig. 3d, the POM solution was well confined between a hydrophilic flat substrate and a superhydrophobic pillar-structured template. With the evaporation of water, interspaces between micropillars served as wetting defects to control the rupture of POM mother liquid, thereby yielding individual microdroplet on the top of each pillar. Subsequently, the microdroplet readily condense into POM microcrystal, generating patterned microcrystal arrays on the flat substrate. Therefore, these results revealed that the combination of hydrophilic flat substrate and superhydrophobic pillar-structured template endowed the POM mother liquid with well-organized rupture and controlled domains, thereby leading to the generation of patterned POM microcrystal arrays.

### Patterning arrays of various POMs on air-grid superwettable surfaces

**Figure 4:**
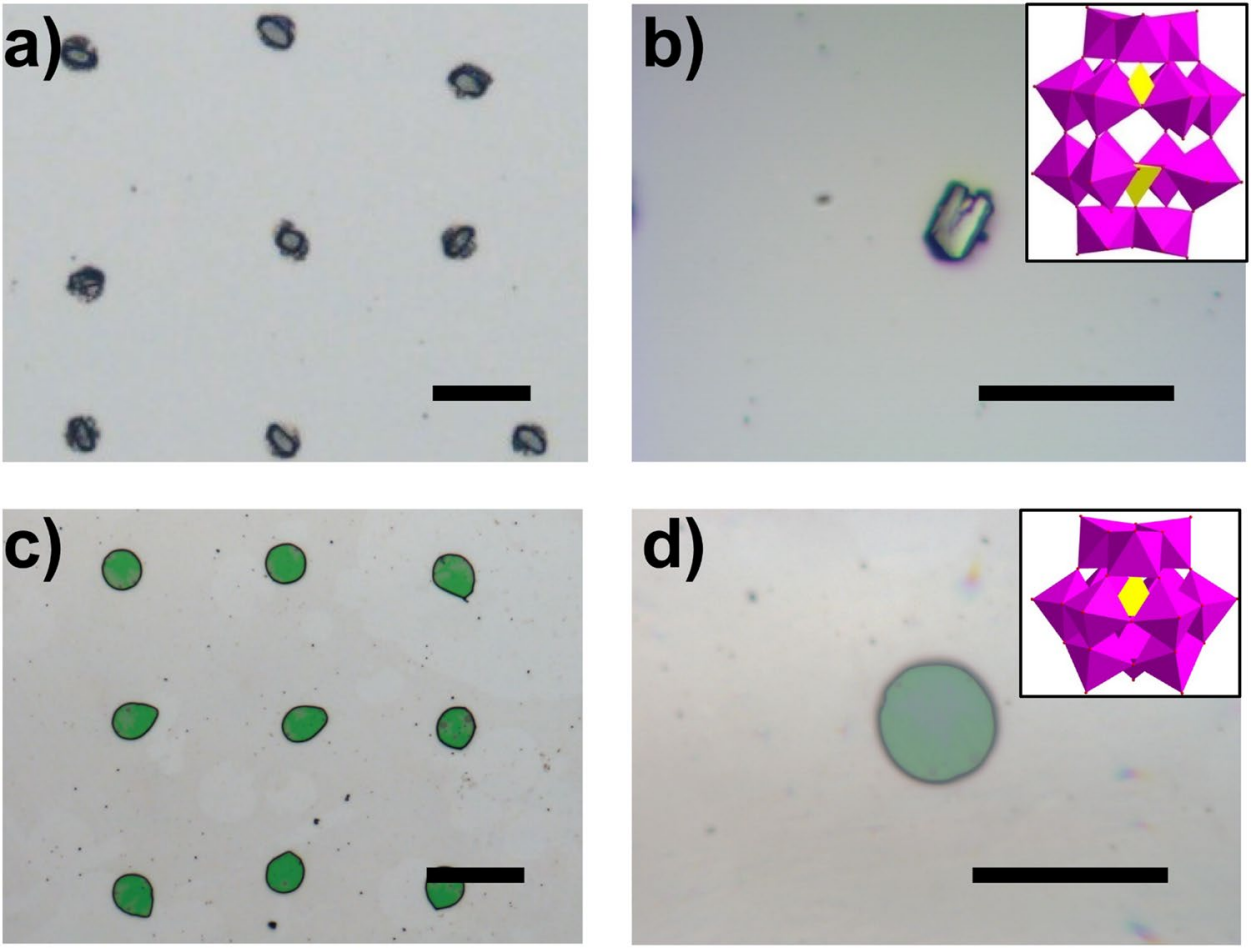
Two typical POM crystal arrays patterned on air-grid superwettable surfaces including (**a,b**) Wells-Dawson-typed K_6_P_2_W_18_O_64_ (denoted as P_2_W_18_) and (**c,d**) Keggin-typed Na_3_PW_12_O_40_ (denoted as PW_12_). Insert images in (**b,d**) show the corresponding crystal structures of P_2_W_18_ and PW_12_.

To further apply this air-grid superwettable surface, several typical POM mother liquid such as Keggin-typed Na_3_PW_12_O_40_ (denoted as PW_12_)^34^ and Wells-Dawson-typed K_6_P_2_W_18_O_62_ (denoted as P_2_W_18_)^35^ were also employed to fabricate POM microcrystal arrays. As shown in the optical images of Fig. 4 and the IR spectra in Fig. S1, patterned P_2_W_18_ and PW_12_ crystal arrays could be well concentrated and arranged from POM mother liquid. Interestingly, we observed that their morphologies of POM microcrystals were entirely different from each other such as rectangle-shaped P_2_W_18_ and round-shaped PW_12_ crystals, which may originate from the crystal habits of these POM building blocks (the insert in Fig. 4b,d). Therefore, these results suggested the universality of the POM patterning method based on the air-grid superwettable surface.

## Conclutions

In conclusion, we have patterned POM-based microcrystal array on air-grid superwettable surface. The size of POM crystal arrays was readily adjustable by tailoring the diameter of pillars or the concentration of POM mother liquid. While the distribution of POM crystal arrays can be controlled by changing the pillar spacing. We envision that this new air-grid surface patterning method based on superhydrophobic templates can be widely employed for patterning POM-based crystal arrays, bringing potential applications in POM-based function materials and devices.

## Methods

### Materials

(100) oriented smooth silicon wafers (n-type) were purchased from GRINM Semiconductor Materials Co., LTD. Pillar-structured silicon substrates were prepared by standard photolithography techniques^36^. In our experiments, we fabricated pillar-structured silicon substrates with adjustable pillar diameters (20, 40 and 60 μm) and pillar periods (10, 20 and 30 μm), constant pillar heights (20 µm) and circular pillar-top shape (Fig. S2). After resist-stripping (Microposit Remover 1165), the substrates were cleaned using ethanol and acetone prior to the chemical modification process. According to previously published procedures, three kinds of typical POMs such as Anderson-structured (NH_4_)_3_[CoMo_6_O_24_H_6_] (denoted as CoMo_6_), Keggin-structured Na_3_PW_12_O_40_ (denoted as PW_12_) and Wells-Dawson-structured K_6_P_2_W_18_O_62_ (denoted as P_2_W_18_) were successfully synthesized^33–35^. Doubly distilled water (>1.82 MΩ cm, MilliQ system) was used. Sulfuric acid (H_2_SO_4_, 95%-98%, AR), Hydrogen peroxide (H_2_O_2_, 30%, AR) were purchased from Beijing Chemical Works and used without further purification. Trichloro-(1 H, 1 H, 2 H, 2H-perfluorooctyl) silane (PFOS) was purchased from Sigma-Aldrich.

### Surface modification of silicon substrates

Firstly, the silicon substrates (i.e., flat substrate and pillar-structured template) were heated in boiling Piranha solution of H_2_SO_4_ and H_2_O_2_ (v/v = 70/30) for 2 h. Then, the substrates were rinsed several times with doubly distilled water and dried by N_2_ flow. Later, these substrates were modified by silanizing with PFOS in a decompression environment at room temperature for 30 min and heated at 80 °C for 8 h. Finally, hydrophobic flat substrate and superhydrophobic pillar-structured substrate could be produced.

### The fabrication of patterned POM crystal arrays

To prepare patterned POM crystal arrays, we employed a sandwich-typed system by the combination of a hydrophilic flat substrate (e.g., silicon), a small droplet of POM mother liquid, and a superhydrophobic pillar-structured silicon substrate as a template. Firstly, a droplet of CoMo6 mother liquid (2 mM, ca. 25 μL) is firstly dropped onto a hydrophilic flat silicon substrate. The superhydrophobic pillar-structured template is covered by the hydrophilic substrate, forming a sandwich-typed system. Then, with gradual evaporation of water in POM liquid at a temperature of 25 °C, regularly individual microdroplets are formatted on the top of each pillar. Later, microcrystal arrays with regular size and restricted position can be patterned from these regularly individual microdroplets with the further evaporation of water. Finally, after gently removing pillar-structured template, the patterned POM crystal arrays could be transferred onto the flat substrate.

## Electronic supplementary material

Supplementary Information

## References

[1] Song YF, Tsunashima R (2012). Recent advances on polyoxometalate-based molecular and composite materials. Chem. Soc. Rev..

[2] Proust A (2012). Functionalization and post-functionalization: a step towards polyoxometalate-based materials. Chem. Soc. Rev..

[3] Miras HN, Vila-Nadal L, Cronin L (2014). Polyoxometalate based open-frameworks (POM-OFs). Chem. Soc. Rev..

[4] Kim M, Chamack M, Geletii YV, Hill CL (2018). Synergetic catalysis of copper and iron in oxidation of reduced keggin heteropolytungstates by dioxygen. Inorg. Chem..

[5] Cai, L. X. *et al*. Water-soluble redox-active cage hosting polyoxometalates for selective desulfurization catalysis. *J. Am. Chem. Soc*. **140**, 4869–4876 (2018). 10.1021/jacs.8b00394 29534562

[6] Anwar N (2018). Redox switching of polyoxometalate-doped polypyrrole films in ionic liquid media. Electrochim. Acta.

[7] Wang M (2018). Sustainable productions of organic acids and their derivatives from biomass *via* selective oxidative cleavage of C-C bond. ACS Catal..

[8] Gao N (2014). Transition-metal-substituted polyoxometalate derivatives as functional anti-amyloid agents for Alzheimer’s disease. Nat. Commun..

[9] Herrmann S, De Matteis L, de la Fuente JM, Mitchell SG, Streb C (2017). Removal of multiple contaminants from water by polyoxometalate supported ionic liquid phases (POM-SILPs). Angew. Chem. Int. Ed..

[10] Martínez-Pérez MJ (2012). Fragmenting gadolinium: mononuclear polyoxometalate-based magnetic coolers for ultra-low temperatures. Adv. Mater..

[11] Han XB (2014). Polyoxometalate-based cobalt-phosphate molecular catalysts for visible light-driven water oxidation. J. Am. Chem. Soc..

[12] Huang P (2012). Self-assembly and photocatalytic properties of polyoxoniobates: {Nb_24_O_72_}, {Nb_32_O_96_}, and {K_12_Nb_96_O_288_} clusters. J. Am. Chem. Soc..

[13] Liu RJ (2016). Enhanced proton and electron reservoir abilities of polyoxometalate grafted on graphene for high-performance hydrogen evolution. Energy Environ. Sci..

[14] Wang LL (2017). A series of novel Anderson-type polyoxometalate-based Mn^II^ complexes constructed from pyridyl-derivatives: assembly, structures, electrochemical and photocatalytic properties. CrystEngComm..

[15] Xu XX, Gao X, Lu TT, Liu XX, Wang XL (2015). Hybrid material based on a coordination-complex-modified polyoxometalate nanorod (CC/POMNR) and PPy: a new visible light activated and highly efficient photocatalyst. J. Mater. Chem. A..

[16] Liu S, Kurth DG, Möhwald H, Volkmer D (2002). A thin-film electrochromic device based on a polyoxometalate cluster. Adv. Mater..

[17] Song YF (2009). Micropatterned surfaces with covalently grafted unsymmetrical polyoxometalate-hybrid clusters lead to selective cell adhesion. J. Am. Chem. Soc..

[18] Huang J, Kim F, Tao AR, Connor S, Yang P (2005). Spontaneous formation of nanoparticle stripe patterns through dewetting. Nat. Mater..

[19] Bai L (2014). Bio-inspired vapor-responsive colloidal photonic crystal patterns by inkjet printing. ACS Nano.

[20] Song W, Veiga DD, Custódio CA, Mano JF (2009). Bioinspired degradable substrates with extreme wettability properties. Adv. Mater..

[21] Feng W, Li L, Du X, Welle A, Levkin PA (2016). Single-step fabrication of high-density microdroplet arrays of low-surface-tension liquids. Adv. Mater..

[22] Li G (2015). Rapid cell patterning induced by differential topography on silica nanofractal substrates. Small.

[23] Lu Y, Lin B, Qin J (2011). Patterned paper as a low-cost, flexible substrate for rapid prototyping of PDMS microdevices via “liquid molding”. Anal. Chem..

[24] Wang S, Liu K, Yao X, Jiang L (2015). Bioinspired surfaces with superwettability: new insight on theory, design, and applications. Chem. Rev..

[25] Chen L, Yang G, Wang S (2012). Air-grid surface patterning provided by superhydrophobic surfaces. Small.

[26] Krumpfer JW, McCarthy TJ (2011). Dip-coating crystallization on a superhydrophobic surface: a million mounted crystals in a 1 cm^2^ array. J. Am. Chem. Soc..

[27] Su B, Wang S, Ma J, Song Y, Jiang L (2011). “Clinging-microdroplet” patterning upon high-adhesion, pillar-structured silicon substrates. Adv. Funct. Mater..

[28] Hatton BD, Aizenberg J (2012). Writing on superhydrophobic nanopost arrays: topographic design for bottom-up assembly. Nano Lett..

[29] Feng J (2016). “Liquid knife” to fabricate patterning single-crystalline perovskite microplates toward high-performance laser arrays. Adv. Mater..

[30] Wu Y (2015). Positioning and joining of organic single-crystalline wires. Nat. Commun..

[31] Su B (2014). A general strategy for assembling nanoparticles in one dimension. Adv. Mater..

[32] Wu Y, Feng J, Su B, Jiang L (2016). 3D dewetting for crystal patterning: toward regular single-crystalline belt arrays and their functionality. Adv. Mater..

[33] Nomiya K, Takahashi T, Shirai T, Miwa M (1987). Anderson-type heteropolyanions of molybdenum (VI) and tungsten (VI). Polyhedron.

[34] Rocchiccioli-Deltcheff C, Fournier M, Franck R, Thouvenot R (1983). Vibrational investigations of polyoxometalates. 2. Evidence for anion-anion interactions in molybdenum (VI) and tungsten (VI) compounds related to the Keggin structure. Inorg. Chem..

[35] Contant R, Klemperer WG, Yaghi O (1998). Potassium octadecatungstodiphosphates (V) and related lacunary compounds. Inorg. Synth..

[36] Linnros J, Badel X, Kleimann P (2006). Macro pore and pillar array formation in silicon by electrochemical etching. Phys Scr.

