# Supplementary material for: Polyoxometalate-based microcrystal arrays patterned on air-grid superwettable surface
Source: Sci Rep. 2018 Sep 17;8:13915. doi: 10.1038/s41598-018-32279-4 (PMC6141463; doi:10.1038/s41598-018-32279-4)
Supplement: Supplementary file 1 — Supplementary Information [file 41598_2018_32279_MOESM1_ESM.docx]

# Polyoxometalate-based microcrystal arrays patterned on air-grid superwettable surface

## Tianzhan Zhang^1,2,†^, Yuefeng Wang^1,3,†^, Jun Bing Fan^1^, Jingxin Meng^1*^, Yangguang Li^4*^, Enbo Wang^4^ & Shutao Wang^1,3*^

[1] CAS Key Laboratory of Bio-inspired Materials and Interfacial Science, CAS Center for Excellence in Nanoscience, Technical Institute of Physics and Chemistry, Chinese Academy of Sciences, Beijing, 100190, P. R. China

[2] College of Material Science and Engineering, Jilin Jianzhu University, Changchun, 130118, P. R. China

[3] University of Chinese Academy of Sciences, Beijing, 100049, P. R. China

[4] Key Laboratory of Polyoxometalate Science of Ministry of Education, Faculty of Chemistry, Northeast Normal University, Changchun, 130024, P. R. China.

† These authors contributed equally.

*Corresponding authors

Jingxin Meng (email: [mengjx628@mail.ipc.ac.cn](mailto:mengjx628@mail.ipc.ac.cn))

Yangguang Li (email: liyg658@nenu.edu.cn)

Shutao Wang (email: stwang@mail.ipc.ac.cn)


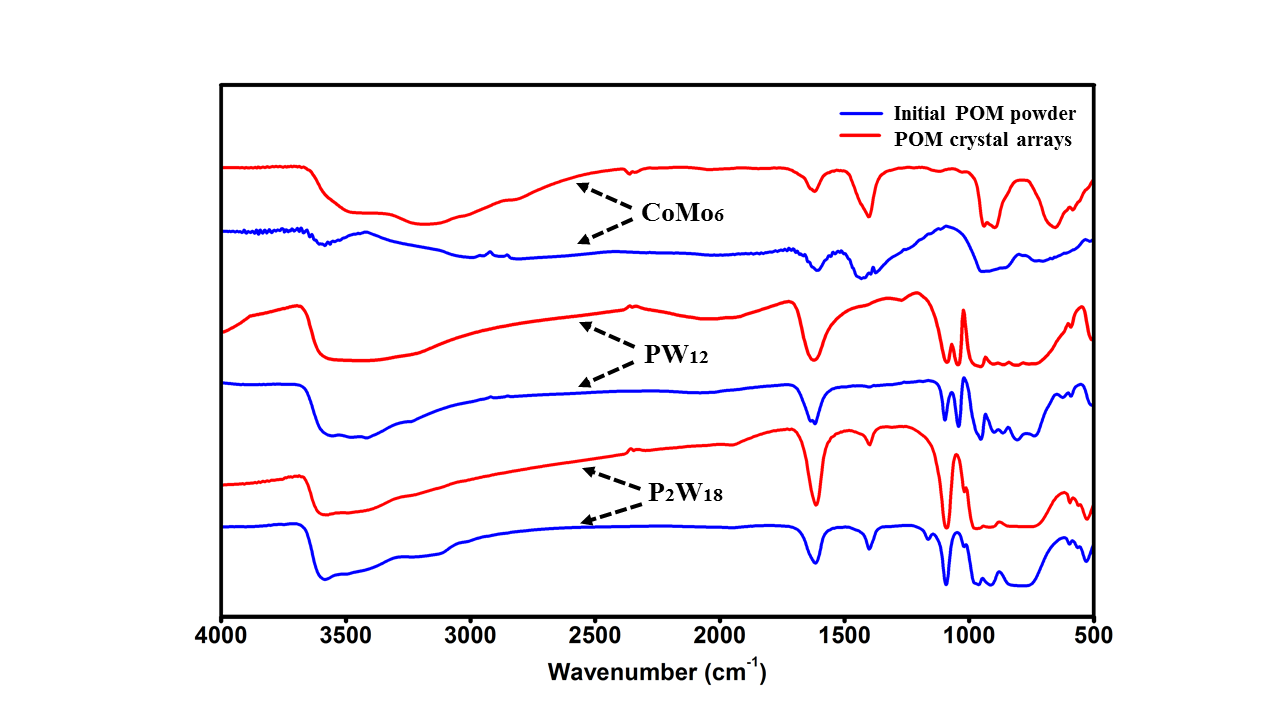


**Figure S1.** The IR spectra of three typical polyoxometalates (POMs) including Anderson-typed (NH_4_)_3_[CoMo_6_O_24_H_6_] (denoted as CoMo_6_), Keggin-typed Na_3_PW_12_O_40_ (denoted as PW_12_) and Wells-Dawson-typed K_6_P_2_W_18_O_62_ (denoted as P_2_W_18_). The characteristic peaks of different POMs indicate the existence of different POMs.

.


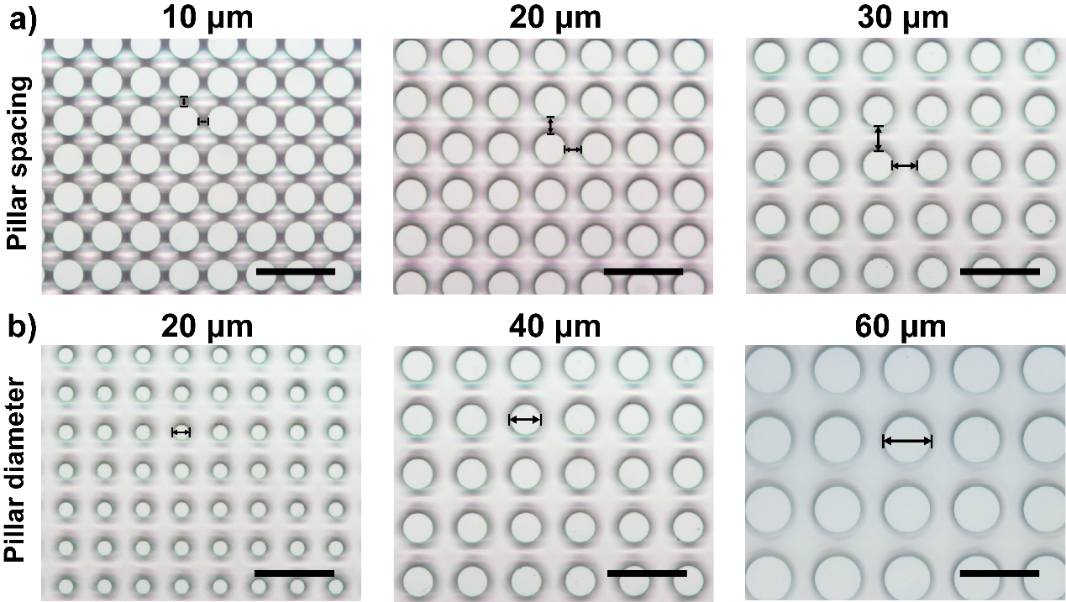


**Figure S2.** The optical images of circular pillar-structured silicon templates for preparing POM crystal arrays. a) The pillar-structured silicon substrates with pillar spacings varying from 10 to 30 µm (pillar diameter: 40 µm; pillar height: 20 µm). b) The pillar-structured silicon substrates with pillar diameters varying from 20 to 60 µm (pillar spacing: 30 µm; pillar height: 20 µm). The scale bars are 100 µm.


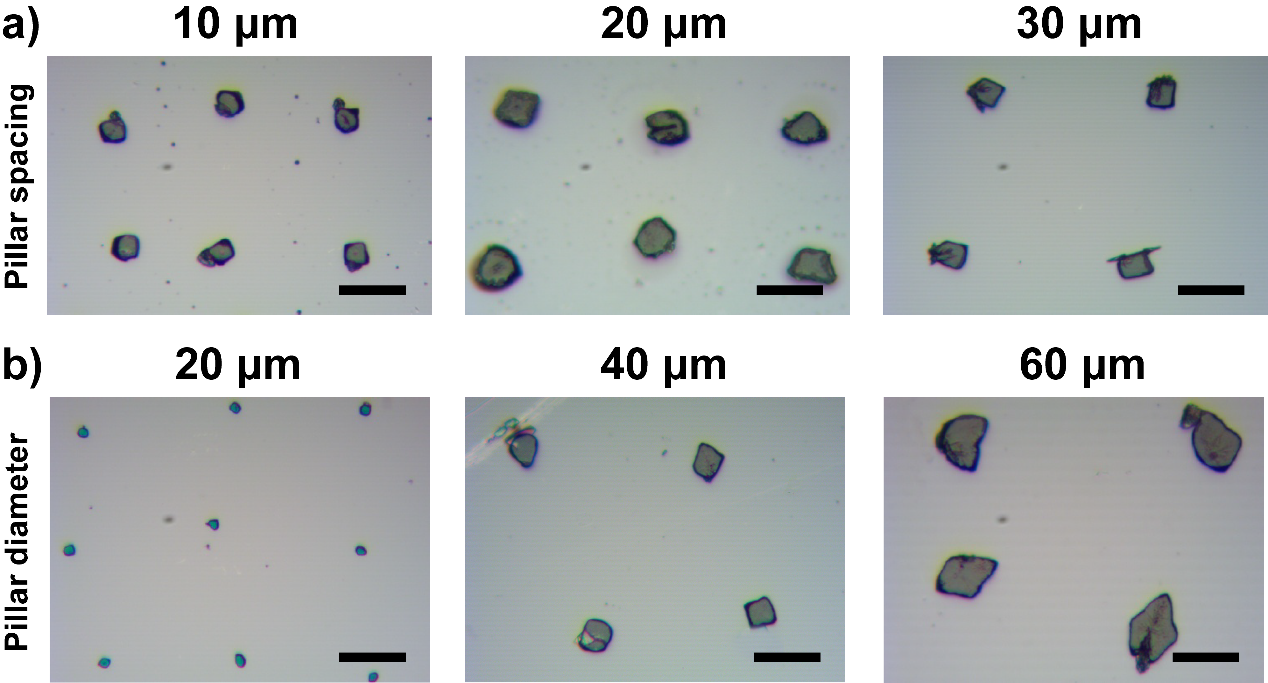


**Figure S3.** The optical images show the influence of pillar-structured template (i.e., pillar spacing and diameter) on patterned POM crystal arrays. a) The pillar spacings have no apparent effect on crystal area but crystal distribution. b) The crystal areas increase with the increment of pillar diameters. The scale bars are 25 µm.


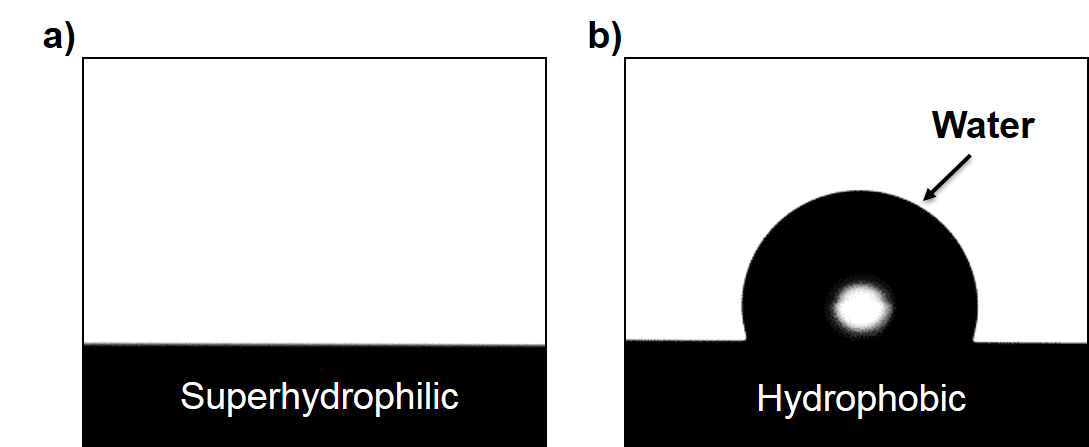


**Figure S4.** The optical images of water droplets (about 2 µL) on two kinds of silicon substrates including a) pillar-structured silicon substrates with superhydrophilic property and b) PFOS-modified flat substrates with hydrophobic property.


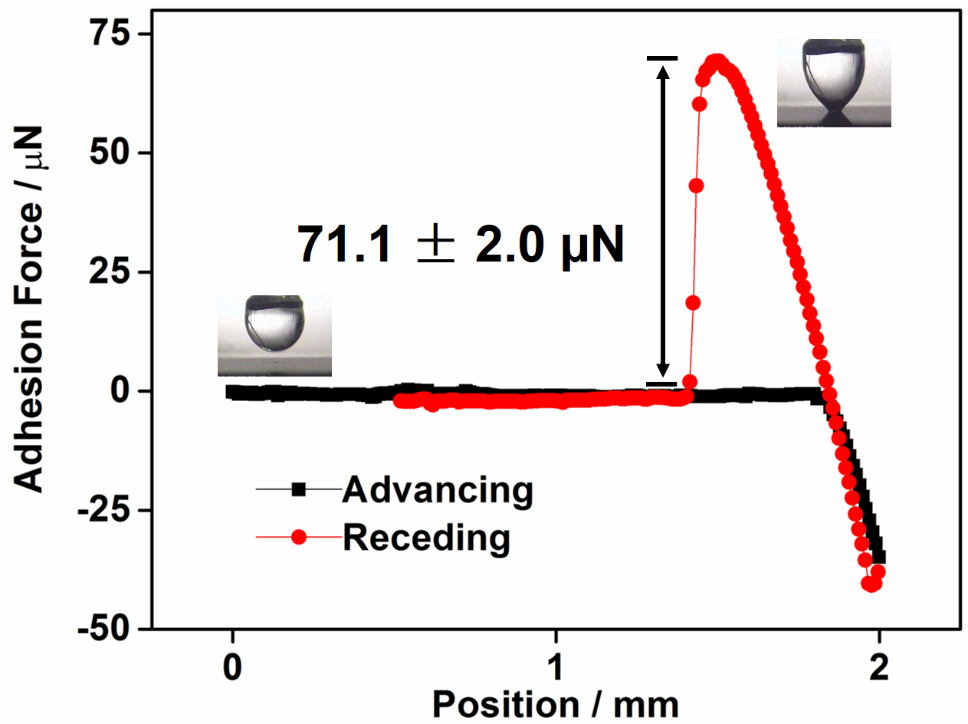


**Figure S5.** The force-distance curves before and after the water droplet was in contact with the superhydrophobic pillar structured template, showing a high water adhesion of the superhydrophobic pillars.
